# Correction to Anti‐acne activity of carnitine salicylate and magnolol through the regulation of exfoliation, lipogenesis, bacterial growth and inflammation

**DOI:** 10.1111/srt.13451

**Published:** 2023-08-23

Kwon, KC, Won, JG, Kim, M‐S, Shin, YW, Park, S‐W, Song, Y‐S. Anti‐acne activity of carnitine salicylate and magnolol through the regulation of exfoliation, lipogenesis, bacterial growth and inflammation. *Skin Res Technol*. 2023; 29:e13406. https://doi.org/10.1111/srt.13406

‘SZ95’ was incorrect and should be changed to ‘sebocyte’ throughout the article.

In page 3, section 2.3 Cell culture, Celprogen, Torrance, CA has been added to the text.

The online version of the article has been corrected.

We apologize for this error.

